# The clinical relevance of surgical specimens for RNA sequencing in lung cancer: a cohort study

**DOI:** 10.3389/fonc.2024.1462519

**Published:** 2024-10-18

**Authors:** Jung Seop Eom, Soo Han Kim, Kyungbin Kim, Ahrong Kim, Hyo Yeong Ahn, Jeongha Mok, Jeong Su Cho, Min Ki Lee, Ju Sun Song, Mi-Hyun Kim

**Affiliations:** ^1^ Department of Internal Medicine, Pusan National University School of Medicine, Busan, Republic of Korea; ^2^ Department of Internal Medicine, Pusan National University Hospital, Busan, Republic of Korea; ^3^ Biomedical Research Institute, Pusan National University Hospital, Busan, Republic of Korea; ^4^ Department of Pathology, Pusan National University Hospital, Busan, Republic of Korea; ^5^ Department of Pathology, Pusan National University School of Medicine, Busan, Republic of Korea; ^6^ Department of Thoracic and Cardiovascular Surgery, Pusan National University School of Medicine, Busan, Republic of Korea; ^7^ Department of Laboratory Medicine, Green Cross (GC) Genome Corporation, Yongin, Republic of Korea

**Keywords:** next-generation sequencing, RNA, surgical procedures, operative, lung neoplasm

## Abstract

**Background:**

Molecular screening using next-generation sequencing (NGS) in the pathologic evaluation of lung cancer is considered the standard in clinical practice; hence, we evaluated the diagnostic yields of various sampling methods for NGS.

**Methods:**

NGS data from patients with lung cancer at the Pusan National University Hospital (Busan, South Korea), admitted October, 2020–April, 2023, was obtained. The sampling methods by which NGS data was obtained were divided into surgical and nonsurgical. Surgical methods included thoracoscopic surgery, surgical biopsy from the metastatic site, and lymph node excisional biopsy, whereas nonsurgical methods included bronchoscopy procedures and medical thoracoscopic biopsy.

**Results:**

In total, we obtained 319 patients’ NGS data:150 (47.0%) and 169 (53.0%) was obtained using surgical and nonsurgical methods, respectively. The overall diagnostic yield of NGS analysis was 97.5% for all samples. There were no significant differences in the success rates of deoxyribonucleic acid sequencing between surgical and nonsurgical sampling methods (98.0% vs. 96.4%, p *=* 0.313). On the other hand, the success rate of ribonucleic acid (RNA) sequencing was significantly lower in the surgical method group (78.0% vs. 92.3%; p < 0.001). Multivariate analysis showed that surgical sampling significantly correlated with RNA sequencing failure (Odd Ratio 4.128, 95% Confidence Interval 1.681–10.133, p *=* 0.002).

**Conclusions:**

Small samples obtained using nonsurgical procedures are suitable for NGS analysis in clinical practice. However, surgical sampling showed a relatively lower success rate for RNA sequencing than nonsurgical sampling. This information may help in the development of protocols to reduce RNA degradation during the surgical process.

## Introduction

Next-generation sequencing (NGS), known also as massively parallel or deep sequencing, describes a deoxyribonucleic acid (DNA)/ribonucleic acid (RNA) sequencing technology (1). NGS technologies were introduced between 2004 and 2006, transforming biomedical inquiries and resulting in a dramatic increase in sequencing data outputs (2, 3). This novel technology has revolutionized our understanding of tumor biology and consequently improved survival over the past decade (4, 5). NGS was mainly used for academic purposes at early development, however, facilitated by decreasing costs and overall increased availability, it has recently been widely implemented in the routine diagnostic workflows at many institutions (6).

NGS has proven to be a valuable strategy for improving the diagnosis, prognosis, and treatment decisions in the field of oncology, including lung cancer. NGS provides a comprehensive analysis of gene mutations, rearrangements, and amplifications in the DNA/RNA-based reactions of targeted genes (3, 5). RNA plays an essential role in numerous biological processes and RNA-based biomolecules hold promise for various applications in oncology (7). Detection of gene fusions and differential expression of known disease-causing transcripts by RNA sequencing represents some of the most immediate opportunities (7–9). Therefore, the ESMO guidelines recommend that NGS be performed on either RNA or DNA if it includes level I fusions in the panel (6). Furthermore, NGS offers information on rare mutations and co-occurring alterations previously unidentifiable through conventional testing, potentially uncovering druggable targets or providing insights into resistance mechanisms (3, 5). To date, current international guidelines strongly recommend molecular testing, including epidermal growth factor receptor (EGFR), anaplastic lymphoma kinase (ALK), kirsten rat sarcoma (KRAS), ROS Proto-Oncogene 1 (ROS1), v-Raf murine sarcoma viral oncogene homolog B1 (BRAF), neurotrophic tyrosine kinase receptor 1/2/3 (NTRK1/2/3), mesenchymal epithelial transition (MET) exon 14 skipping, rearranged during transfection proto-oncogene (RET), and Erb-B2 receptor tyrosine kinase 2 (ERBB2) (human epidermal growth factor receptor 2 (HER2)) mutations, with broader molecular profiling to identify rare driver mutations for which effective drugs may already be available (5, 10, 11).

However, the successful application of NGS technology in routine practice faces several challenges. Many technical and cost-associated considerations play a role in the decision-making processes (4). Notably, the most reliable sampling method in clinical practice and the quantity and quality of the obtained tumor tissue samples for NGS has not been widely evaluated.

Here, we report the yield of NGS analysis based on various sampling methods, and the unreliability of surgical specimens for RNA sequencing in lung cancer.

## Materials and methods

### Patients

NGS data from patients with lung cancer at the Pusan National University Hospital (a university-affiliated, tertiary referral hospital in Busan, Republic of Korea), during October, 2020– April, 2023, was obtained. Some of the patients in the current study have already been included in our previous study (12). Actionable mutations in the NGS reports were defined as available or potential targets for anticancer treatment, including *EGFR, ALK, ROS-1, KRAS (G12C), BRAF (V600E), NTRK, MET, RET, and ERBB2* mutations. Co-mutations were also identified in NGS reports. This study was approved by the Institutional Review Board (IRB) of Pusan National University Hospital (IRB no. 2305-028-127). This study was conducted in accordance with the principles of the Declaration of Helsinki. The requirement for informed consent was waived by the IRB of Pusan National University Hospital because of the retrospective nature of the study and because the analysis used anonymous clinical data.

### Sampling methods for NGS analysis

The sampling methods for the NGS analysis were divided into two groups: surgical and nonsurgical. Surgical methods included thoracoscopic surgery, surgical biopsy from the metastatic site, and lymph node excisional biopsy. Nonsurgical methods included bronchoscopy procedures [transbronchial lung biopsy (TBB)], endobronchial ultrasound-guided transbronchial needle aspiration (EBUS-TBNA)) and medical thoracoscopic biopsy. The decision regarding the tumor sampling modality was based on the anatomical location of the primary lung cancer, involvement of the mediastinal lymph nodes, applicability of the procedure, and performance status of the patient.

### Nucleic acid extraction and NGS analysis

Briefly, from our previous study (12), DNA and RNA were extracted from formalin-fixed and paraffin-embedded (FFPE) samples using a total DNA/RNA extraction kit (RecoverALL, Thermo Fisher Scientific, Waltham, MA, USA) following the manufacturer’s protocol. Targeted NGS was performed using the Oncomine Comprehensive Assay Plus (OCA-Plus, Thermo Fischer Scientific, Waltham, MA, USA). OCA-Plus covers 501 cancer-associated genes and allows the detection of single-nucleotide variants, multiple-nucleotide variants, small insertions/deletions, amplifications, and fusions. In addition, the OCA-Plus includes microsatellite instability and tumor mutational burden assays. NGS library preparation for OCA-Plus using the extracted DNA and synthesized cDNA was performed using the Ion AmpliSeq Library Preparation on the IonChef System protocol (Thermo Fisher Scientific, Waltham, MA, USA) following the manufacturer’s instructions. The deamination reaction implemented in OCA-Plus was conducted using Uracil-DNA glycosylase-heat labile (Thermo Fisher Scientific, Waltham, MA, USA) prior to polymerase chain reaction (PCR) amplification. Sequencing was performed on the IonTorrent S5 XL platform (Thermo Fisher Scientific) with an Ion 550 Chip Kit (Thermo Fisher Scientific, Waltham, MA, USA), according to the manufacturer’s instructions. RNA libraries were prepared from 20 ng RNA which were mixed with two primer pools and the AmpliSeq HiFi Master Mix and transferred to a PCR cycler (SimpliAmp™ Thermal Cycler, Life Technologies, Thermo Fisher Scientific, Waltham, MA, USA). After the end of the PCR reaction, RNA primer end sequences were partially digested using FuPa reagent, followed by the ligation of barcoded sequencing adapters (Ion Xpress Barcode Adapters; Life Technologies, Thermo Fisher Scientific, Waltham, MA, USA). The final libraries were purified using Celemics MagBeads (Celemics, Seoul, South Korea) and quantified using quantitative PCR.

### Statistical analysis

Continuous variables were analyzed using Student’s *t*-test, and dichotomous variables were analyzed using χ^2^ or Fisher’s exact test. The success rate of each sampling method was analyzed using Fisher’s exact test. All *p*-values were two-sided, and statistical significance was set at *p* < 0.05. Multivariate analysis of factors related to RNA sequencing failure was performed using logistic regression. All statistical analyses were performed using the IBM SPSS Statistics Software for Windows (version 25.0; IBM Corp., Armonk, NY, USA).

## Results

### Patient characteristics

A total of 319 patients were enrolled in this study. The clinical characteristics of the patients are summarized in **Table 1**.

**Table 1:** Patient characteristics.

	All patients (n = 319)	Surgical (n = 150)	Non-surgical (n = 169)	P value
Median age (range)	69 (39–89)	68 (40–83)	69 (39–89)	0.143
Sex, n (%)				0.002
Male	209 (65.5)	85 (56.7)	124 (73.4)	
Female	110 (34.5)	65 (43.3)	45 (26.6)	
Histology. n (%)				<0.001
ADC	212 (66.5)	121 (80.7)	91 (53.8)	
SCC	69 (21.6)	19 (12.7)	50 (29.6)	
SCLC	21 (6.6)	4 (2.7)	17 (10.1)	
NSCLC, NOS	12 (3.8)	2 (1.3)	10 (5.9)	
Other^*^	5 (1.5)	4 (2.6)	1 (0.6)	
Stage, n (%)				<0.001
I	46 (14.4)	45 (30.3)	1 (0.6)	
II	33 (10.3)	31 (20.7)	2 (1.2)	
III	58 (18.2)	29 (19.3)	29 (17.2)	
IV	182 (57.1)	45 (30.0)	137 (81.1)	

Other^*^: adenosquamous (n = 3), large cell carcinoma (n = 2).

ADC, adenocarcinoma; SCC, squamous cell carcinoma; SCLC, small-cell lung cancer; NSCLC, non-small-cell lung cancer; NOS, non-small cell lung cancer not otherwise specified.

The median age was 69 years (range, 39–89 years), and 209 patients (65.5%) were men. Adenocarcinoma was the most common pathological subtype (n = 212, 66.5%), followed by squamous cell carcinoma (SCC) (n = 69, 21.6%). According to the American Joint Committee on Cancer staging system 8^th^ edition, 14.4% (n = 46) of the patients were stage I, 10.3% (n = 33) were stage II, 18.2% (n =58) were stage III, and 57.1% (n = 182) were stage IV. Patients who underwent nonsurgical methods had more non-adenocarcinoma histology (p < 0.001) and advanced stages (p < 0.001). A total of 254 samples (79.6%) were from treatment-naïve patients and 65 samples (20.4%) were from previously treated patients.

Of the 311 patients’ samples available for NGS, 148 (47.5%) harbored actionable mutations. Details of the actionable mutations reported at the time of sample collection are shown in **Table 2**.

**Table 2:** Actionable mutation status in patients determined using next-generation sequencing.

n (%)	n = 148 (47.5%, of 311)	Treatment-naïve (n = 109)	Re-biopsy (n = 39)
*EGFR*			
Common (19del, L858R)	71 (48.0)	55 (50.5)	16 (41.0)
Exon 20 insertion	5 (3.4)	5 (4.6)	0 (0)
Common+T790M	4 (2.7)	1 (0.9)	3 (7.7)
L861Q	1 (0.6)	0 (0)	1 (2.6)
S768I + G724S	1 (0.6)	1 (0.9)	0 (0)
*EGFR* + other^*^	13 (8.8)	7 (6.4)	6 (15.4)
*ALK* fusion	5 (3.4)	5 (4.6)	0 (0)
*ROS1* fusion	4 (2.7)	4 (3.7)	0 (0)
*KRAS G12C*	10 (6.8)	8 (7.3)	2 (5.1)
*BRAF V600E*	0 (0)	0 (0)	0 (0)
*NTKR*	0 (0)	0 (0)	0 (0)
*MET*			
Exon 14 skipping	9 (6.1)	7 (6.4)	2 (5.1)
Amplification	14 (9.5)	11 (10.1)	3 (7.7)
* RET* fusion	5 (3.4)	2 (1.8)	3 (7.7)
* HER2*	6 (4.0)	3 (2.8)	3 (7.7)

^*^Other: MET amplification (n = 7), MET 14 skipping (n = 2), ALK fusion (n = 2), BRAF V600E (n = 1), and T790M+MET amplification (n = 1)

EGFR, epidermal growth factor receptor; ALK, anaplastic lymphoma kinase; ROS1, ROS proto-oncogene 1; KRAS, kirsten rat sarcoma; BRAF, v-Raf murine sarcoma viral oncogene homolog B1; NTKR, neurotrophic tyrosine kinase receptor; MET, mesenchymal epithelial transition; RET, rearranged during transfection proto-oncogene; HER2, human epidermal growth factor receptor 2

### Comparison of the sampling methods for NGS analysis

The details of the sampling methods used for the 319 patients are presented in **Table 3**. Among the specimens, 150 (47.0%) and 169 (53.0%) were obtained using surgical and nonsurgical methods, respectively. Among all specimens, eight samples (2.5%) showed suboptimal quality for NGS analysis, resulting in an overall NGS yield of 97.5% (311/319 patients). Of these eight samples, six were obtained using nonsurgical methods (five from TBB and one from medical thoracoscopic biopsy), and two from surgical lymph node excisional biopsy. Seven of these eight samples provided sufficient materials for histological diagnosis; however, the quantity was insufficient for NGS sequencing. In one case, only necrotic material was obtained, preventing any histological diagnosis from being made.

**Table 3:** Comparison of the sampling methods (nonsurgical vs surgical).

	Surgical method (n = 150)			Non-surgical method (n = 169)			P value
	Thoracoscopic surgery	Surgical biopsy from metastatic sites	Lymph node excisional biopsy	TBB	EBUS-TBNA	Medical thoracoscopic biopsy	
n, (%)	123 (38.6)	17 (5.3)	10 (3.1)	137 (42.9)	13 (4.1)	19 (6.0)	
DNA (ng/μL) Median, range	53.2 (3.76–271.0)	76.8 (11.4–120.0)	58.2 (39.4–120.0)	39.7 (3.70–148.0)	58.2 (5.68–120.0)	37.7 (19.1–120.0)	0.009
RNA (ng/μL) Median, range	170.0 (0–788.0)	102.0 (7.04–606.0)	134.0 (19.0–256.0)	72.1 (5.42–440.0)	10.8 (0–120.0)	181.0 (22.6–994.0)	<0.001
NGS success rate (%)	100	100	80.0	96.4	100	94.7	0.003
DNA-based NGS (%)	99.2	100	80.0	96.4	100	94.7	0.036
RNA-based NGS (%)	76.4	94.1	70.0	93.4	76.9	89.5	<0.001

NGS, next generation sequencing; TBB, transbronchial lung biopsy; EBUS-TBNA, endobronchial ultrasound-guided transbronchial needle aspiration.

The median yields of DNA and RNA quantities obtained from each sampling method were significantly higher in the surgical method group than in the nonsurgical method group (for DNA, median 53.6 (range, 3.76–271.00) and 40.2 (range, 3.7–148.0) ng/μL, p < 0.001) and (for RNA, median 120.0 (range, 0–788.0) and 75.8 (range, 0–994.0) ng/μL, p < 0.001). The success rates of NGS were 97.2% for DNA sequencing and 85.6% for RNA sequencing. There were no significant differences in the success rates of DNA sequencing between surgical and nonsurgical sampling methods (98.0% vs. 96.4%, p *=* 0.313). However, the success rate of RNA sequencing was significantly lower in the surgical method group (78.0% vs. 92.3%; p < 0.001). The NGS success rates for each method are listed in **Table 3**.

### Clinical factors associated with the risk of RNA sequencing failure

A total of 46 cases (14.4%) showed suboptimal RNA sequencing quality. Thirty-three samples (71.7%) were obtained using surgical methods, and 13 samples (28.9%) were obtained using nonsurgical methods. The clinical characteristics of the patients are shown in **Table 4**.

**Table 4:** Clinical characteristics of patients exhibiting RNA sequencing failure.

	n = 46
Median age (range)	68 (39–87)
Sex, n (%)	
Male	35 (76.1)
female	11 (23.9)
Histology, n (%)	
ADC	33 (71.7)
SCC SCLC	5 (10.9) 3 (6.5)
NSCLC, NOS	1 (2.2)
Other^*^	4 (8.6)
Stage, n (%)	
I	8 (17.4)
II	7 (15.2)
III	14 (30.4)
IV	17 (37.0)
Sampling methods, n (%)	
Surgical	33 (71.7)
Non-surgical	13 (28.9)
Median yield of DNA (ng/μl) (range)	33.2 (3.76–271.0)
Median yield of RNA (ng/μl) (range)	112.0 (0–640.0)

Other^*^: adenosquamous (n = 3), large cell carcinoma (n = 1)

ADC, adenocarcinoma; SCC, squamous cell carcinoma; SCLC, small-cell lung cancer; NSCLC, non-small-cell lung cancer; NOS, non-small cell lung cancer not otherwise specified

There were no statistically significant differences in the clinical characteristics between patients who showed RNA sequencing failure and those who did not. Univariate analysis revealed that advanced stage and surgical sampling methods were significantly correlated with the risk of RNA sequencing failure. Multivariate analysis showed that only surgical sampling methods were significantly correlated with RNA sequencing failure (Odd Ratio (OR) 4.128, 95% Confidence Interval (CI) 1.681–10.133, p *=* 0.002) (**Table 5**).

**Table 5:** Analysis of the clinical factors for RNA sequencing failure.

	Univariate		Multivariate	
	OR (95% CI)	P value	OR (95% CI)	P value
Age (yr)				
<69	1	Reference		
≥70	1.607 (0.841–3.072)	0.149	1.480 (0.755–2.901)	0.254
Sex				
Male	1	Reference		
Female	1.810 (0.880–3.723)	0.103		
Histology				
ADC	1	Reference		
Non-ADC	0.750 (0.743–4.544)	0.412	0.911 (0.431–1.922)	0.806
Stage				
Non-advanced	1	Reference		
Advanced	2.100 (1.111–3.970)	0.021	1.337 (0.559–3.195)	0.514
Time for NGS				
Treatment-naïve	1	Reference		
Re-biopsy	1.838 (0.743–4.544)	0.182	2.199 (0.794–6.086)	0.129
Sampling methods				
Non-surgical	1	Reference		
Surgical	3.385 (1.706–6.715)	<0.001	4.128 (1.681–10.133)	0.002

OR, odds ratio; 95% CI, 95% confidence interval; ADC, adenocarcinoma

## Discussion

In this study, we report the diagnostic yield of NGS analysis based on various sampling methods and the NGS results in clinical practice. The overall diagnostic yield of NGS analysis was 97.5% for all the sampling methods. The success rates of DNA and RNA sequencing were 97.2% and 85.6%, respectively.

Among the nonsurgical methods, TBB was the most commonly used method for NGS (42.9%). We observed a relatively high success rate for both DNA and RNA sequencing (96.4% and 93.4%, respectively) using TBB. Furuya et al. summarized and reported the feasibility of using TBB for NGS analysis in several previous studies (13). The DNA sequencing success rate was 71.3%–100%, and the RNA sequencing success rate was 64%–84.4% (13–17). The authors explained that the use of fresh frozen tissue was one reason for the relatively high success rate compared with previous studies. Fresh frozen tissue is the preferred sample for analyzing gene mutations because of its superiority in preserving nucleic acid quality (18). However, fresh frozen tissue is often not available in clinical practice, due to a complicated protocol and relatively high cost (18, 19). In this study, we demonstrated a high success rate of NGS analysis using small FFPE samples obtained using TBB. Moreover, EBUS-TBNA showed comparably high NGS success rates (100%), despite the relatively lower extracted yield of nucleic acids. There have been concerns that EBUS-TBNA is limited in terms of the volume that can be used to obtain tumor tissue (20). However, our findings suggest that respiratory physicians could choose among several bronchoscopy procedures for NGS analysis depending on the tumor localization.

Unexpectedly, the success rate of RNA sequencing in surgical specimens was much lower than that in nonsurgical specimens, despite the larger amount of RNA extracted. Multivariate analysis also showed that the surgical method was significantly associated with RNA sequencing failure. In particular, thoracoscopic surgery specimens showed an RNA sequencing success rate of 76.4%. Several hypotheses have been proposed to explain the phenomenon. The first pertains to the tissue fixation time. Traditional methods for storing histological and cytological specimens for future use in molecular assays consist of either snap-freezing with subsequent cryopreservation, or collection in a fixative or preservation solution, most frequently in the form of FFPE, which is the standard preservation procedure for routine clinical diagnostics in pathology laboratories (19). Formalin fixation is commonly used in routine organizational examinations (21–23). Formalin is cost-effective, safe for human exposure, easy to use, and versatile, making it suitable for almost all staining purposes, except for some specific cases (21–23). As tissue permeability is approximately 1 mm/h at room temperature, the fixation duration varies depending on the type of fixative and the tissue size. Generally, small biopsy specimens require several hours to 12 hours, whereas tissues exceeding 5 mm in thickness are fixed for approximately 24 hours (23, 24). Therefore, the amount of RNA degradation may be higher in surgical specimens than in small specimens obtained using various nonsurgical methods because of the longer fixation time of the surgical specimens.

Another factor may be the effects of time delay and surgical antiseptics (24). In contrast to DNA, RNA is extremely unstable and degrades easily. Because most clinical specimens are obtained during surgical operations, doctors have limited time for protective storage of these samples. This leads to substantial degradation of RNA. Wilcox et al. have reported the effects of time delay and surgical antiseptics on RNA isolated from human and rodent peripheral nerves (25). They found that time delays between surgical liberation and cryopreservation significantly decreased RNA concentrations. In addition, the detrimental effect of antiseptic surgical reagents such as chlorhexidine and iodine on RNA yield was confirmed in a rodent model, where the RNA yield was 8.3-fold lower than that in non-exposed samples. Moreover, in this study, the time from the NGS prescription date to the start date in the surgical method group was significantly longer than that in the nonsurgical method group (median 3 days (range 0–34, vs 1 d (range 0–10), p < 0.001). These factors might cause a significantly low success rate in RNA sequencing using surgical methods. Therefore, our results suggest that samples for NGS analysis should be processed separately from the entire surgically harvested specimen. However, further studies are required to confirm this phenomenon.

This study had several limitations. First, this was a single-center study that encompassed challenges in generalization, the risk of selection bias, limited diversity, and site-specific factors, potentially compromising external validity and replicability. However, with a relatively large sample size and our institution’s status as a referral center in the region, we contend that these factors mitigate these limitations to some extent. Second, the exact fixation time for each sample is unclear. Undoubtedly, surgical specimens require more fixation time than small specimens obtained using nonsurgical methods because of their larger volume; however, the exact time for sample fixation has not been recorded. Third, we need to perform additional experimental studies are required to further investigate the effects of time delay and exposure to antiseptic agents.

In conclusion, this study reported the success rates of NGS analysis based on various sampling methods and results. Small FFPE samples obtained using bronchoscopy procedures are suitable for NGS analysis in clinical practice. These findings suggest that bronchoscopy is a reasonable diagnostic tool for NGS, particularly for patients with advanced lung cancer. However, surgical specimens showed a relatively lower success rate for RNA sequencing than nonsurgical specimens. This information may help in the development of protocols to reduce RNA degradation during the surgical process

## Data Availability

The datasets used and/or analyzed during the current study are available from the corresponding author upon reasonable request.
